# Do US Ambient Air Lead Levels Have a Significant Impact on Childhood Blood Lead Levels: Results of a National Study

**DOI:** 10.1155/2013/278042

**Published:** 2013-08-01

**Authors:** LuAnn L. Brink, Evelyn O. Talbott, Ravi K. Sharma, Gary M. Marsh, Wen Chi Wu, Judith R. Rager, Heather M. Strosnider

**Affiliations:** ^1^Department of Epidemiology, University of Pittsburgh Graduate School of Public Health, 130 DeSoto Street, Pittsburgh, PA 15261, USA; ^2^University of Pittsburgh Academic Partner of Excellence in Public Health Tracking, USA; ^3^Department of Behavioral and Community Health Sciences, University of Pittsburgh Graduate School of Public Health, 130 DeSoto Street, Pittsburgh, PA 15261, USA; ^4^Department of Biostatistics, University of Pittsburgh Graduate School of Public Health, 130 DeSoto Street, Pittsburgh, PA 15261, USA; ^5^Center for Occupational Biostatistics and Epidemiology, University of Pittsburgh, USA; ^6^Division of Environmental Hazards and Health Effects, Centers for Disease Control and Prevention, Environmental Health Tracking Branch, 1600 Clifton Road, Atlanta, GA 30333, USA

## Abstract

*Introduction*. Although lead paint and leaded gasoline have not been used in the US for thirty years, thousands of US children continue to have blood lead levels (BLLs) of concern. *Methods*. We investigated the potential association of modeled air lead levels and BLLs ≥ 10 **μ**g/dL using a large CDC database with BLLs on children aged 0–3 years. Percent of children with BLLs ≥ 10 **μ**g/dL (2000–2007) by county and proportion of pre-50 housing and SES variables were merged with the US EPA's National Air Toxics Assessment (NATA) modeled air lead data. *Results*. The proportion with BLL ≥ 10 **μ**g/dL was 1.24% in the highest air lead counties, and the proportion with BLL ≥ 10 **μ**g/dL was 0.36% in the lowest air lead counties, resulting in a crude prevalence ratio of 3.4. Further analysis using multivariate negative binomial regression revealed that NATA lead was a significant predictor of % BLL ≥ 10 **μ**g/dL after controlling for percent pre-l950 housing, percent rural, and percent black. A geospatial regression revealed that air lead, percent older housing, and poverty were all significant predictors of % BLL ≥ 10 **μ**g/dL. *Conclusions*. More emphasis should be given to potential sources of ambient air lead near residential areas.

## 1. Introduction 

Although blood lead levels (BLL) in US children have been dramatically reduced over the past 40 years [1], lead poisoning events continue to occur. The Lead-Based Paint Poisoning Prevention Act legislation was passed in 1971, and by 1978 the use of lead-based paint in residential housing was banned [2]. Regulations phasing out lead in gasoline were implemented in 1973. The elimination of lead from these two sources has resulted in a dramatic reduction in BLLs. However, there are still subgroups of children in both urban and rural areas with high BLLs. Data evaluated from 26 states that are part of the Centers for Disease Control (CDC) Childhood Lead Poisoning Prevention Program (CLPPP) and have data available on the CDC Environmental Public Health Tracking Network (Tracking Network) revealed that almost 95,000 children between 0 and 3 years of age had confirmed blood lead ≥10 *μ*g/dL from 2000 to 2007 with an estimated 7,000 children in 2006 alone.

In 2002, over 9,000 industrial sites reported lead releases to the US Environmental Protection Agency (EPA) Toxic Release Inventory (TRI) [3]. Lead is one of the EPA [4] Criteria Air Pollutants that have been established as harmful to either human health or the environment. The current National Ambient Air Quality Standards for lead are 0.15 *μ*g/m^3^ for a rolling 3-month average and 1.5 *μ*g/m^3^ for a quarterly average. The contribution of industrial sources to BLLs has been studied in select industrial communities [5–8]; however, a rigorous systematic evaluation of ambient air lead and its possible contribution to blood lead in US children has not been done at the county level or census tract level.

Aside from many occupational studies [9–12] that link air and dust lead from lead smelters, battery reclamation, and mining jobs with high BLLs among workers, there have been very few studies that specifically examined the contributions of lead in air and lead in soil to childhood BLLs [13–20]. Although a great deal has been accomplished to minimize the exposure to lead paint in the US, little research has been done to quantify the modest but consistently higher childhood BLLs among those living in areas near industrial sources emitting lead. 

A review article by Levin et al. [19] estimates that lead paint and dust from household sources account for up to 70% of BLL ≥ 10 *μ*g/dL in US children, but more than 30% of these children do not have exposure to lead paint in their home. Most recently, Miranda et al. [20] evaluated children's BLLs by proximity to airports with planes using avgas, a leaded fuel used in small aircraft. The highest BLLs were in children within 500 m of an airport, but there was an impact within 1000 m. The US EPA [21] has determined that exposure to lead from avgas may be through inhalation directly from air or ingestion from soil after the lead has deposited.

Although BLLs in the US have fallen considerably with the removal of lead in gasoline and the considerable efforts of the Healthy Homes and Lead Poisoning Prevention Branch (HHLPPB) at CDC, Housing and Urban Development (HUD), and state and local health departments, there are counties in the US where as many as 10% of screened children have a BLL ≥ 10 *μ*g/dL. Clusters of higher BLLs raise considerable concern that we have not completed the task. The Tracking Network houses over five million childhood blood lead results by county and the proportion of children per county who have confirmed BLLs ≥ 10 *μ*g/dL. Given the availability of these data and the information on air lead levels by census tract and county from the USEPA National-Scale Air Toxics Assessment (NATA), we evaluated the linkage of these two comprehensive databases to determine the overall relationship at the ecological level as the first step in understanding the role of ambient air lead to childhood blood lead poisoning. The purpose of this investigation is to utilize air lead exposure and biomonitoring data on childhood blood lead screening available on the Tracking Network to develop methods to characterize populations who may be at higher risk of lead poisoning. 

## 2. Methods

The Tracking Network uses data collected by state and local childhood lead poisoning prevention programs to track childhood BLL. It is recommended that high-risk children are screened for blood lead between the ages of 0–3. The results of these tests are provided to state and local public health programs, funded in part by HHLPPB. The state and local departments then share information with the HHLPPB to compile a national database. To conduct a comprehensive nation-wide evaluation of the impact of air lead on children's BLLs, the University of Pittsburgh requested childhood blood lead data from the HHLPPB for 1644 (52%) of the 3220 US counties. 

Some states mandatorily test all children aged 0–3, while others test only a “targeted” population most at risk for lead poisoning, usually those who receive Medicaid or live in older housing. Data included the number of children ages 0–3 found to have BLL ≥ 10 *μ*g/dL and the number of children who had blood lead testing for each year 2000–2007 by county. Summary information, including the total number of children tested per county for all years available, was calculated using SPSS version 18. For the following analysis, only those counties with at least 50 children tested over the eight year period were selected (*n* = 1508 counties). The excluded counties tested an average of four children per year and had an average population of 29,372. For 2007, only 77 of the 1508 (5%) had reported lead data at the time of the data request. Therefore, 2007 data is also not presented. Annual percent high and a summary total ≥10 *μ*g/dL, total tested, and percent ≥10 *μ*g/dL per county were calculated using SPSS. 

### 2.1. NATA Data (2002 and 2005)

Every three years since 1996, EPA has compiled a national-scale air toxics assessment (NATA). The assessment is a state-of-the-science tool that provides estimates of the concentrations, exposures, and broad estimates of the risk from breathing air toxics. In 2002, NATA evaluated 180 of the 187 Air Toxics, including lead and lead compounds. The 2005 NATA estimated 177 of the 187 air toxics. The NATA assessment involves compiling a national air toxics emissions inventory of outdoor stationary and mobile sources (National Emissions Inventory, NEI). These sources include major stationary sources, for example, large waste incinerators and factories; area and other sources, for example, dry cleaners, small manufacturers, and wildfires; and both on road and nonroad mobile sources, for example, cars, trucks, planes, and boats. 

We conducted a sensitivity analysis of the validity of NATA estimates. The EPA monitors to which we compared the NATA estimates exist for these regulatory purposes. Under the Clean Air Act, EPA sets and reviews national air quality standards for lead. We compared the average yearly measurement from these lead monitors to the NATA concentration estimate in the census tract in which the monitor sits. The over 200 lead monitors measure concentrations of lead throughout the country. Quartiles of NATA concentrations and quartiles of monitor readings were compared using weighted Kappa statistics. 

We used modeled ambient concentrations of lead and lead compounds at the county level for the 2005 National-Scale Air Toxic Assessment (NATA downloaded from the EPA website) [22]. There were notable improvements from the 2002 to 2005 NATA estimates, which include inclusion of 19,000 airports as point sources, onroad and nonroad inventories were updated, and the state-of-the-art Community Multi-Scale Air Modeling (CMAQ) model was used. Because of these improvements, the 2005 model was used. These air lead levels were merged by FIPS code to the county of interest and its corresponding blood lead testing data.

### 2.2. 2000 Census Data

Additional county-level census variables that were downloaded to explore their predictive value for childhood BLLs include percentage of older (pre-1950) housing, percent living in poverty, and racial makeup of the county. The three types of data (health, EPA NATA, and Census) were merged by FIPS code using SPSS.

In addition, counties were classified into the following based upon the NCHS Urban Rural Classification Scheme Methodology [23]. They were classified into (1) Central counties of a metro area with more than a million people, (2) fringe counties of a metro area of more than a million people, (3) counties in a metro area of 250,000 to 999,999 people, (4) counties in a metro area of 50,000 to 24,999 people, (5) micropolitan counties (20,000 to 49,999, adjacent to a metro area), and (6) noncore counties, with a population between 20,000 and 49,999 and not adjacent to a metro area or a population under 20,000 whether or not they are adjacent to a metro area. For this analysis, classification no. 1 is “Urban,” no. 2–5 are “Suburban,” and no. 6 is “Rural.”

### 2.3. Data Analysis

First, we compared the mean proportion of children with high BLL who reside in the top 10% (highest decile) of high air lead counties with the proportion with high BLL who reside in the counties with the lowest air lead (lowest decile). Second, a nonparametric correlation matrix was created and analyzed to determine the degree of interrelationship between percent pre-1950 housing, poverty, race, rural (as defined by the National Center for Health Statistics), and percent of children with BLL ≥ 10 *μ*g/dL. 

In order to utilize the relationship between the proportion of children with high BLL and demographic/census variables such as NATA air lead estimate in 2005, percent pre-1950 housing, rural, African Americans, and percent of population at less than 185% of poverty, we first applied multiple linear regression. However, our response variable and model did not meet the assumptions of normality and linearity. Therefore, we used Box-Cox transformation with parameter 0.297 (indicating a cube root transformation) to improve normality and linearity. The Box-Cox generalizes power transformations as

(1) $$\begin{array}{c} y_{i}^{\theta }=\frac{\left(y_{i}^{\theta }-1\right)}{\theta },\;\text{where}\;\;\theta \neq 0,\\ y_{i}^{\theta }=\ln\left(y_{i}\right),\;\text{where}\;\;\theta =0 \end{array}$$

 
*θ* = 2 square transformation
 
*θ* = 1 (no transformation)
 
*θ* = 1/2 square root transformation
 
*θ* = 1/3 cube root transformation
 
*θ* = 0 log transformation
 
*θ* = −1 reciprocal transformation.

### 2.4. Negative Binomial Regression

As an alternative to using the proportion of children with high BLL as the dependent variable, we modeled the counts of children with high BLL among total number of children tested per county from 2000 to 2007 (offsetting the number of children tested) using Poisson regression. Because the Poisson regression models indicated overdispersion (variance exceeding the mean) our final models were based on negative binomial regression. These models were used to investigate the dependence of counts of children with high blood levels on the NATA air lead estimates without and with adjustment for the demographic and census variables noted above.

The negative binomial log linear regression for the rate is

(2) $$\begin{array}{c} \log\left(\frac{\mu }{n}\right)=\alpha +\beta x,\\ \log\left(\mu \right)-\log\left(n\right)=\alpha +\beta x,\\ \mu =ne^{\alpha +\beta x}, \end{array}$$

where *μ* is the expected value of counts (ex: the expected value of children with high BLLs), *n* is index of the time or space (ex: total number of children tested per county), and −log⁡(*n*) is an adjustment term which is referred to as an offset, and each subject may have a different value of *n*.

### 2.5. Spatial Linear Regression

Counties in our sample are spatially related to each other by virtue of being spatial neighbor and therefore subject to common underlying exposures. Spatial autocorrelation (or dependence) is measured in terms of first-order (i.e., only adjacent counties) contiguity where the dependent variable or error term for each county is correlated with observations for the dependent variable or error term at contiguous locations. Aspatial forms of regression are applied to all of the data within a county, quantifying global relationships between variables, assuming no spatial correlation. These results serve as our baseline analysis. If the relationship across space between the independent variable(s) and dependent variables does not change, and error terms are independent between counties, the aspatial regression would provide our final results.

However, as we detected significant spatial correlation among residuals from the aspatial regression, we next considered spatial regression allowing correlated residual terms. However, as the dependent variable did not approximate the normal distribution, the cube root of the term percent ≥10 *μ*g/dL was taken, as spatial regression uses a multiple linear regression. We begin with general formulation of spatial regression model defined as a spatial lag model and also referred to as the spatial autoregressive model. The spatial lag model is expressed as follows:

(3) $$\begin{array}{c} Y=\rho Wy+X\beta +\varepsilon ,\\ \varepsilon \;\sim N\left(0,\sigma^{2}I_{n}\right), \end{array}$$

where *Y* is an *n* by 1 vector of observations on the dependent variable (percent with BLL ≥ 10 *μ*g/dL), *X* is the matrix of county characteristics, *W*_*y*_ is the row-standardized spatial weight matrix, and the parameter *ρ* is a coefficient on the spatial lag of the dependent variable. “*Wy*” captures the extent to which BLLs are affected by levels in contiguous counties. *β* denotes the vector of coefficients associated with the independent variables and *ε* denotes the error terms (25). The blood level data by county is only available for selected counties in the US, and as such the boundary file has “islands” where no data are available for BLL. We therefore cannot calculate a contiguity matrix that expresses the neighborhood structure. We are therefore limited to using a distance matrix for a spatial weight matrix using the centroids of each county with BLL data. This avoids problems of “islands” in the data.

ArcGIS version 10 provides the polygons representing all the counties in our sample. GeoDa (a freely downloadable software package) version 0.9.5-i by Luc Anselin was used to develop and estimate the spatial lag.

## 3. Results

**Figure 1:**
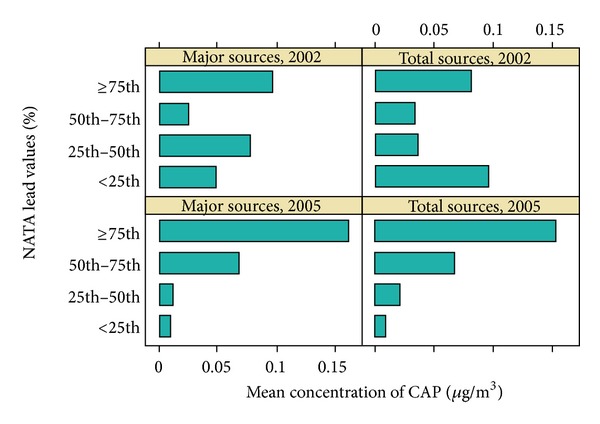
Mean concentrations of Pb from criteria air pollutant lead monitors by NATA percentile.

The sensitivity analysis of lead monitor readings and NATA air lead estimates indicated a correlation of *R* = 0.91 (*P* < .001) and a Kappa of 0.424, which indicates good agreement (26). See Figure 1.

In 2000, the proportion of children <36 months of age with BLL ≥ 10 *μ*g/dL ranged from 8.89% (IL) to 0.42% (KY, WA). In 2006, this ranged from 2.45% (CT) to 0.42% (LA). The proportion of children with BLL ≥ 10 *μ*g/dL in general fell from 2000 to 2006, among most states for example, Michigan from 4.06% in 2000 to 0.98% in 2006 and Ohio from 3.5% in 2000 to 1.21% in 2006 and among those tested in Illinois 8.89% were over 10 *μ*g/dL in 2000 and 3.52% in 2004. Clearly, pockets of children at high risk for childhood lead poisoning remain across the country. See Table 1.

**Figure 2:**
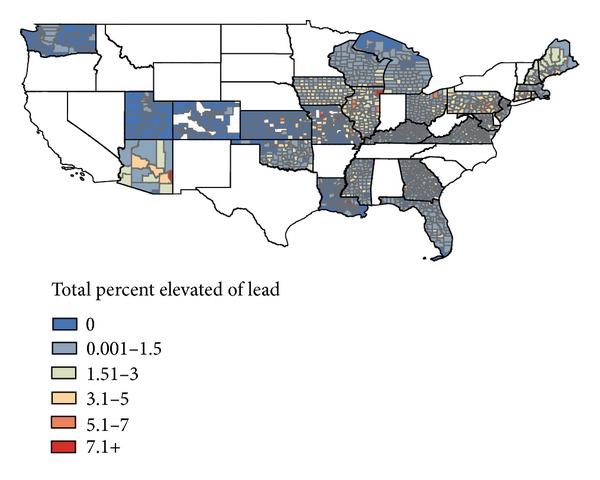
Cumulative percentage of children with BLL ≥ 10 *μ*g/dL by county, 2000–2007.

When the counties that were included in the sample were stratified into highest and lowest deciles of air lead, we found that the highest ten percent of air lead included those counties with total concentrations greater than 0.00297 *μ*g/m^3^ while the counties with the lowest decile of air lead had concentrations below 0.000526 *μ*g/m^3^. The proportion with BLL ≥ 10 *μ*g/dL was 1.24% in the highest air lead counties, and the proportion with BLL ≥ 10 *μ*g/dL was 0.36% in the lowest air lead counties, resulting in a crude prevalence ratio of 3.4. See Figure 2.

The counties included in this analysis had a range of poverty (from 2.6 to 41.1%), minority (from 0.3 to 86.5% Black), and older housing (from 0.8 to 66.7%). Population within a county ranged from less than 2000 to over five million. As these have been established as important independent predictors of lead burden, these were controlled for in the analysis. See Table 2.

BLL ≥ 10 *μ*g/dL by county were moderately correlated with NATA lead (*r* = 0.15, *P* < .001) and older housing (*r* = 0.37, *P* < .001) by county. The census covariates (poverty, rural, Black) were all highly correlated with NATA exposure estimates (*P* < .001). 

A negative binomial regression was considered with blood lead data as the dependent (predicted) variable and air lead, percent pre-1950 housing, and percent rural as the predictive/independent variables. The advantage of a negative binomial regression is that it weights each county by number of children tested. The result of univariate binomial regression analysis shows that county-level NATA for 2002, NATA for 2005, percentage of pre-1950 housing per county, rural classification of the county, and county-specific percentage below poverty are statistically significant while percentage black is borderline significant (*P* = 0.074). See Table 3. The correlation among these explanatory variables has been examined and percent black and percent below poverty have a positive, significant correlation (0.54) whereas others appear to have a relatively weak relationship. In addition, the collinearity diagnostics indicates that none of explanatory variables in a multiple regression model are highly correlated. 

In the *univariate* negative binomial regression, proportion black and mandatory or targeted testing in a county were not significant. In *multivariate* negative binomial regression, NATA modeled air lead was a significant predictor of childhood blood lead (% ≥ 10 *μ*g/dL) after adjusting for % pre-l950 housing, rural classification, and percent of black children by county. Because NATA 2005 was a slightly better predictor in the univariate model, these NATA estimates were entered into the multivariate model. Percent below poverty was not significant in *multivariate* analysis.

The negative binomial loglinear regression for the rate is

(4) log⁡(μn)=α+βx,log⁡(μ)−log⁡(n)=α+βx,μ=neα+βx,log⁡(μ)=β0+β1∗NATA05+β2∗pre50+β3∗suburban+β4∗rural+β5∗black+log⁡⁡(tottest).

The percent change in the relative risk of total number of BLL ≥ 10 *μ*g/dL increases 36% for every 0.01 *μ*g/m^3^ increase in NATA air lead value, 5% for every unit increase in % pre-1950 housing, and 1% for every unit increase in % black people per county. Children living in suburban and rural areas decrease their relative risk by 14% and 34% relative to those living in the urban areas. Children living in mandatory counties are 36% less likely to have BLL ≥ 10 *μ*g/dL than those living in targeted counties. See Table 4.

Finally, the results of a geospatial regression further solidified the relationship between childhood blood lead and air lead and older housing. The cube root transformation of the proportion of children with a BLL ≥ 10 *μ*g/dL was chosen as the dependent variable to satisfy normality. The analysis revealed that poverty, pre-1950 housing, and air lead levels were all significant predictors of BLL ≥ 10 *μ*g/dL (Table 5). The *R* ^2^ value was 0.420 (*P* < .001), and air lead, percent older housing, percent black, and urban/suburban were all significant predictors (*P* < .001). The coefficient of NATA05 indicates that the cube root of percent elevated is predicted to increase 72.8 when NATA05 is increased by one and decrease by 0.23 when urban/suburban/rural jumps to the next category. See Table 5.

After accounting for spatial autocorrelation of the residuals, R-squared increased to 0.39. All independent variables remain statistically significant. The likelihood ratio test of spatial error dependence is significant. Therefore, we conclude that a spatial lag model is fitted better than the OLS model using cube-root transformation of percent elevated BLLs. 

## 4. Discussion

It is well known that a child's home environment has the clearest relationship with a child's BLL [1]. Childhood BLL have decreased with the removal of lead from paint and gasoline. There remain, however, 250,000 US children annually who exhibit BLL ≥ 10 *μ*g/dL which can affect their mental and physical health. This study was the first to use a national data base on childhood BLL over an eight-year period to link proportion children with a BLL ≥ 10 *μ*g/dL in children with NATA 2005-modeled air lead as well as percent pre-1950 housing, race, and poverty. While many of the primary risks have been removed from our environment, the task of eliminating childhood lead poisoning [19] has not been completed. Air lead has been identified [13–20] as a contributor to childhood BLL in smaller scale studies. These studies were conducted in distinct metro areas and near significant sources of air lead and soil lead such as nearby smelters and lead producing areas. This national assessment of the contribution of air lead to childhood BLLs, controlling for known risk factors such as older housing and poverty levels, will help to quantify the increase in BLL that may be attributed to differences in air lead throughout the US. 

Our research group recognizes that there are limitations to epidemiologic methods. While ecologic studies are generally quicker and less cumbersome to conduct, they are prone to the ecologic fallacy and other forms of bias, which can arise from the absence of detailed information on the joint distribution of exposure and outcome within the groups under study. As such, ecologic studies are most useful as hypothesis generating and not hypothesis testing investigations, often providing important clues to occupational and environmental determinants of disease. For example, in the mid-1970s a series of ecologic studies conducted by the US National Cancer Institute implicated industrial factors in the development of various malignant diseases [24].

There are differences in childhood blood lead testing frequency that may influence this study as well. Differences exist between states—even counties within the same state—regarding blood lead testing policies. Typically, there are targeted and mandatory testing policies. We applied “targeted” or “mandatory” to each county within a state based upon the state's policies as stated in 2010. However, these policies may change annually, as public health funding dictates. In addition, some counties may have targeted towns or neighborhoods with known housing or lead industry issues, elevating the likelihood of a child having a high level, thereby increasing the proportion of children with BLL ≥ 10 *μ*g/dL. A sensitivity analysis of proportion tested in a sample of targeted and mandatory counties showed that the proportion tested in mandatory counties was double that tested in targeted counties. However, a full complement of data was not available due to privacy concerns. Targeted testing typically focuses on high-risk children, often identified as those on Medicaid or through additional screening questions. Mandatory testing policies indicate that all children should be tested before age three. However, the consistency of the findings among the univariate and multivariate negative binomial as well as the linear spatial regression model results which predict percent BLL ≥ 10 *μ*g/dL (Tables 3–5) strengthens the conclusion that there is a relationship between ambient air lead levels and percent BLL ≥ 10 *μ*g/dL in younger children. 

In addition, many of the modest effects we observed were deemed statistically significant due to large sample size. Our exposure estimates are static points in time (NATA 2005 and census 2000). For the pollutant of interest for this investigation (lead), NATA average concentrations for 2002 and 2005 were similar [25]. The degree of concordance between NATA and CAPs improved from 2002 to 2005. The average difference between county-level NATA 2002 and NATA 2005 estimates of air lead is 0.0002, indicating that the overall difference is estimates of concentration of air lead that is small. The NATA county-level correlation coefficient for the 2002 and 2005 assessment years was 0.91 (*P* < .0001). However, NATA lead estimates are known to be an underestimation of air lead levels. 

The results of our evaluation of the relationship of air lead and BLLs at the county level for the US indicate that there may remain a significant relationship between ambient air lead and childhood BLL. The proportion with BLL ≥ 10 *μ*g/dL was 1.24% in the highest air lead counties, and the proportion with BLL ≥ 10 *μ*g/dL was 0.36% in the lowest air lead counties, resulting in a crude prevalence ratio of 3.4. This combined with the fact that the percent change in the relative risk of total number of BLL ≥ 10 *μ*g/dL increases 36% for every 0.01 *μ*g/m^3^ increase in NATA air lead value, after controlling for older housing, and rural indicates that there is a significant association between modeled air lead concentration and proportion of children with BLL ≥ 10 *μ*g/dL among those screened in US counties. Further work should be carried out at a more refined geographic level with individual level data to the extent possible to more fully understand the potential contribution of ambient air lead and its concomitant risk from dust inhalation of lead in small children. Proximity to sources of lead emissions should be evaluated as a possible factor to include when identifying children for targeted testing and when evaluating the home environment of a child with BLL ≥ 10 *μ*g/dL.

## Figures and Tables

**Table 1:** Percentage of children with BLL ≥ 10 *μ*g/dL by state and year, 2000–2006.*

State	2000	2001	2002	2003	2004	2005	2006	Universal testing
US	**1.55**	**1.39**	**1.32**	**1.07**	**0.97**	**0.75**	**0.70**	
AZ	2.29	2.13	4.95	1.25	0.47	—	—	Targeted
CO	—	—	—	—	—	—	—	Mandatory
CT	4.50	4.20	3.95	3.90	3.27	2.99	2.45	Mandatory
FL	0.96	0.86	0.50	0.36	0.30	—	—	Targeted
GA	0.76	0.62	0.53	0.31	0.22	0.18	—	Targeted
IA	2.21	1.98	1.87	1.69	1.53	1.12	0.89	Mandatory
IL	8.89	6.75	5.00	4.26	3.52	—	—	Targeted
KS	2.50	1.64	1.10	1.03	1.21	0.62	0.46	Mandatory
KY	0.42	0.57	0.59	0.39	0.41	0.30	0.53	Targeted
LA	—	—	1.09	0.78	0.46	0.37	0.42	Mandatory
MD	1.98	1.20	0.98	0.77	0.54	0.32	0.23	Targeted
ME	2.13	1.91	1.74	1.62	1.80	1.30	—	Targeted
MA	—	—	1.13	1.05	0.92	0.83	0.66	Mandatory
MI	4.06	2.91	2.27	1.89	1.65	1.16	0.98	Targeted
MS	1.88	1.21	0.93	0.78	0.67	0.59	0.54	Targeted
MO	2.27	1.92	1.64	1.40	1.08	0.92	0.89	Targeted
NH	2.10	2.27	2.18	1.91	1.38	1.45	—	Targeted
NJ	0.90	1.25	1.59	1.47	1.19	1.03	0.84	Mandatory
OH	3.53	2.72	2.21	1.98	1.65	1.37	1.21	Targeted
OK	1.13	0.94	0.96	0.75	0.80	0.73	—	Targeted
PA	7.04	6.52	4.99	3.38	2.63	2.52	1.95	Targeted
RI	5.27	3.56	3.07	2.71	2.14	—	—	Mandatory
UT	0.36	0.23	0.19	0.66	0.61	0.58	0.76	Targeted
VA	1.06	0.73	0.71	0.83	0.77	0.64	0.44	Targeted
WA	0.42	0.49	0.41	0.49	0.29	0.23	0.17	Targeted
WI	2.64	2.47	2.12	1.91	1.60	1.25	1.20	Targeted

—: Data not available.

*Data provided by CDC Tracking (2/2011).

**Table 2:** Descriptive statistics of 1508 counties with 50 or more children tested for blood lead.

	Percent below poverty	Percent rural	Percent Black	Percent pre-50	Population	NATA 2005
Mean	13.6	56.8	10.6	25.7	97366	0.0013
Min	2.6	0	0.26	0.78	1844	0.0005
Max	41.13	100	86.5	66.7	5376741	0.0149

**Table 3:** Results of univariate negative binomial regressions predicting number of children with BLL ≥ 10 *μ*g/dL by county.

Univariate negative binomial regression		
	Coef.	95% CI
NATA02	66.897*	(35.0818, 98.7209)
NATA05	99.736*	(56.7037, 142.768)
% pre50 housing	0.035*	(0.0320, 0.0385)
Urban (ref)	—	
Suburban	−0.689*	(−1.0317, −0.3466)
Rural	−0.803*	(−1.1508, −0.4551)
% Black	−0.003	(−0.0063, 0.00029)
Targeted/mandatory	0.033	(−0.1035, 0.1697)
% below poverty	−0.019*	(−0.0268, −0.0098)

*P < 0.0001.

**Table 4:** Multivariate negative binomial regression using robust variance estimation predicting number of children with BLL ≥ 10 *μ*g/dL per US county.

Negative binomial regression				
Variables	Relative risk	Robust SE	*P* value	95% CI
NATA*	1.36	0.176	0.017	(1.06, 1.75)
% pre50 housing	1.05	0.002	0.000	(1.04, 1.05)
Urban-rural				
Urban	(reference)			
Suburban	0.86	0.135	0.337	(0.63, 1.17)
Rural	0.66	0.110	0.013	(0.48, 0.92)
% Black	1.01	0.002	0.000	(1.00, 1.01)
Targeted/mandatory testing	0.64	0.041	0.000	(0.56, 0.72)

Note: the predictor of NATA05 air lead was scaled to 100∗ (the original NATA05 air lead).

**Table 5:** Results of spatial lag regression.

Spatial lag regression				
Variables	*β*	SE	*Z* value	*P* value
NATA05	50.83515	10.4221	4.8776	<0.001
% pre50 housing	0.0125	0.00113	11.0852	<0.001
Urban-rural	0.0972	0.0293	3.3193	<0.001
% below poverty	0.0107	0.0022	4.9392	<0.001
Targeted/mandatory testing	−0.2067	0.0347	−5.953	<0.001

*R*-squared: 0.386367				
Sq. Correlation: —				
Sigma-square: 0.219				
S.E of regression: 0.4681				
Log likelihood: −1010.7				
Akaike info criterion: 2035.4				
Schwarz criterion: 2072.62				

## Abbreviations

BLL: Blood lead level
CDC: Centers for Disease Control
CLPP: Childhood lead prevention program
EPA: Environmental Protection Agency
EPHTN: Environmental Public Health Tracking Network
FIPS: Federal information processing standard
HHLPPB: Healthy Homes and Lead Poisoning Prevention Branch
NATA: National Scale Air Toxics Assessment
NEI: National Emissions Inventory
TRI: Toxic Release Inventory
US: United States.
